# Editorial: MSC-derived exosomes in tissue regeneration

**DOI:** 10.3389/fcell.2023.1293109

**Published:** 2023-09-25

**Authors:** Xin-Ming Chen, Xiaodan Wang, Zongliu Hou

**Affiliations:** ^1^ Kolling Institute of Medical Research, Royal North Shore Hospital, St Leonards, NSW, Australia; ^2^ Renal Medicine, The University of Sydney, Darlington, NSW, Australia; ^3^ Central Laboratory of Kunming Yan’an Hospital, Kunming Medical University, Kunming, Yunnan, China; ^4^ Key Laboratory of Tumor Immunological Prevention and Treatment of Yunnan Province, China

**Keywords:** exosome, stem cell, tissue regeneration, tissue injury and regeneration, exosome engineering

Tissue regeneration is a process of renewal, restoration and regrowth of injured or damaged organs and tissues, this repair process includes regeneration of epithelial tissue, fibrous tissue, cartilage tissue, bone tissue, blood vessels, muscle tissue, and nerve tissue, etc. To date, various biomedical technologies such as organ transplantation, tissue engineering, stem cell therapy, miRNA treatment, nanomedicine, organoids, and 3D bio-printing have been applied in the study of regenerative medicine. Among them, stem cell-based tissue regeneration in wound healing, bone disorders, liver fibrosis, kidney fibrosis, cardiovascular fibrosis and neurological disorders has been widely reported and reviewed (Iaquinta et al., 2019; Gubert et al., 2021; Liu et al., 2021; Sivandzade and Cucullo, 2021; Liu et al., 2022; Lukomskyj et al., 2022). For example, bone marrow mesenchymal stem cells (BMSCs) were reported to increase epidermal autophagy and the rate of re-epithelialization, and lead to accelerated healing in diabetic wounds (Shi et al., 2022). Human pluripotent stem cell-derived cartilaginous organoids promote scaffold-free healing of critical size long bone defects (Tam et al., 2021). Adipose-derived stem cell transplantation attenuates inflammation and promotes liver regeneration after ischemia-reperfusion and hemihepatectomy in Swine (Jiao et al., 2019). BMSCs and their conditioned medium attenuate fibrosis in an irreversible model of unilateral ureteral obstruction (da Silva et al., 2015) and adipose-derived stem cells ameliorate renal interstitial fibrosis through inhibition of EMT and inflammatory response via TGF-β1 signaling pathway (Song et al., 2017). MSCs reverse diabetic nephropathy disease via Lipoxin A4 by targeting transforming growth factor β (TGF-β)/smad pathway and proinflammatory cytokines (Bai et al., 2019). MSCs were used to enhance the functionality and therapeutic efficacy of cell-based therapy for cardiovascular disease (Yun and Lee, 2019). MSCs were used as a multimodal treatment for nervous system diseases (Badyra et al., 2020).

Although stem cells have emerged as promising sources for regenerative medicine, there are potential safety concerns, risks and disadvantages in their clinical use such as tumorigenesis, lack of availability, survival time and unknown long-term effects. MSCs are commonly used in cellular therapy trials for immunomodulation and regenerative medicine (Squillaro et al., 2016; Galipeau and Sensebe, 2018), however, one of the key mechanisms of MSC efficacy is considered to be derived from their paracrine activity. MSC-derived exosomes are enriched with therapeutic miRNAs, mRNAs, cytokines, lipids, and growth factors. It has been reported that MSC released extracellular vesicle exosomes (30–150 nm in diameter) may act as paracrine mediators between MSCs and target cells (Heldring et al., 2015; Mendt et al., 2018). MSC-derived exosomes can recapitulate the biological activity of MSCs and may serve as an alternative to whole cell therapy (Lou et al., 2017; Bagno et al., 2018). Numerous *in vivo* studies have demonstrated that the therapeutic benefit of MSCs is principally orchestrated by the paracrine secretion of a broad repertoire of growth factors, chemokines, and cytokines (Deng et al., 2015; Wang et al., 2015; Yao et al., 2015). Due to their natural involvement in the intercellular exchange of biomolecules, exosomes hold great potential as a novel biotherapy. The use of MSC-derived exosomes may provide considerable advantages over their counterpart live cells due to a higher safety profile, lower immunogenicity, and the inability to directly form tumors directly (Liew et al., 2017). Therefore, MSC-derived exosomes represent a novel cell-free therapy with compelling advantages over parent MSCs as there is no risk of tumor formation and lower immunogenicity (Mendt et al., 2019; Yin et al., 2019; Joo et al., 2020).

Recently published articles in this journal have updated the progress in *MSC-derived exosomes in tissue regeneration*. Huang et al. have reported that engineering MSC-derived extracellular vesicles (EV) with miR-424 promoted bone repair through targeting the BMP2 cascade. The presented results showed that the engineered MSC-derived EV enhanced osteoinductive function by activating SMADl/5/8 phosphorylation and enhanced bone repair *in vivo*. Based on these results it was concluded that miRNA-based EV engineering could be a promising approach for regenerative medicine. Dong et al. have reported that adipose tissue-derived small EVs (sEV-AT) modulate macrophages to improve the homing of adipocyte precursors and endothelial cells in adipose tissue regeneration. In this study, sEV-AT increased macrophage infiltration significantly, which was followed by improving homing of adipocyte precursors (APs) and endothelial cells (ECs), and sEV-AT-pretreated macrophages improved the migration of APs and ECs, accompanied by the increase of chemokines (MCP-1, SDF-1, VEGF, and FGF) and the activation of NF-kB signaling pathway. As suggested, sEV-AT might regulate the secretion of chemokines via activating the NF-kB signaling pathway to improve homing of APs and ECs and facilitate adipose tissue regeneration Dong et al. Zhao et al. have summarized the therapeutic applications of modified MSC-exosomes in wound healing and skin regeneration in the review article. In this review article, Zhao et al. have comprehensively reviewed recent studies of MSC-exosomes and bioengineering modified MSC-exosomes in skin wound healing and regeneration. It was concluded that MSC-exosomes could provide an alternative option for wound healing. However, further research warrants to a largely increase in the availability of modified MSC-exosomes for future clinical applications in wound healing and skin regeneration. Another study reported by Huang et al. has demonstrated that MSC-derived exosomes ameliorate ischemia/reperfusion induced acute kidney injury in a porcine model. This study has shown that human umbilical cord mesenchymal stem cell (hUC-MSCs)-derived exosomes significantly improved renal function impairment and reduced apoptosis and necroptosis induced by Ischemia/Reperfusion-induced Acute Kidney Injury (I/R-AKI); these exosomes inhibited the expression of some pro-inflammatory cytokines/chemokines, decreased infiltration of macrophages into the injured kidneys and suppressed the phosphorylation of nuclear factor-KB. Interestingly, hUC-MSC exosomes could promote the proliferation of renal tubular cells, angiogenesis and upregulation of Klotho and BMP7 Huang et al. Based on this study, it was concluded that hUC-MSC derived exosomes may be a promising biological therapy for AKI in a clinic.

Among the various exosome-based therapies, MSC-derived exosomes have been well reported and shown the advantage and promising role in regenerative medicine due to their diverse applicability. It was well documented that MSC-derived exosomes modulate various pathophysiological processes such as cell proliferation, migration, angiogenesis, chondrogenesis, neurogenesis, anti-apoptosis, anti-scarring, anti-inflammation, and immunomodulation (Lai et al., 2015; Pashoutan Sarvar et al., 2016; Wu et al., 2018). To date, most reports about *MSC-derived exosomes in tissue regeneration*s were from *in vitro* and animal studies. The clinical studies in the area are very limited. Thus, clinical trials to use MSC-derived exosomes in various tissue regenerations are essential.
