# Corrigendum: Editorial: MSC-derived exosomes in tissue regeneration

**DOI:** 10.3389/fcell.2023.1302578

**Published:** 2023-10-03

**Authors:** Xin-Ming Chen, Xiaodan Wang, Zongliu Hou

**Affiliations:** ^1^ Kolling Institute of Medical Research, Royal North Shore Hospital, Australia; ^2^ The University of Sydney, Australia; ^3^ Kunming Medical University, China; ^4^ Key Laboratory of Tumor Immunological Prevention and Treatment of Yunnan Province, China

**Keywords:** stem cells, exosomes, tissue regeneration, regenerative medicine, tissue injury

In the published article, there was an error regarding the **affiliations** for “Xiaodan Wang and Zongliu Hou.” As well as having **affiliation** 3, they should also have “^4^ *Key Laboratory of Tumor Immunological Prevention and Treatment of Yunnan Province, China”*.

The authors apologize for this error and state that this does not change the scientific conclusions of the article in any way. The original article has been updated.

